# Data for rate versus rhythm control strategy on stroke and mortality in patients with atrial fibrillation

**DOI:** 10.1016/j.dib.2018.08.199

**Published:** 2018-09-06

**Authors:** Chi-Jen Weng, Cheng-Hung Li, Ying-Chieh Liao, Che-Chen Lin, Jiunn-Cherng Lin, Shih-Lin Chang, Chu-Pin Lo, Kuo-Ching Huang, Jin-Long Huang, Ching-Heng Lin, Yu-Cheng Hsieh, Tsu-Juey Wu

**Affiliations:** a Cardiovascular Center, Taichung Veterans General Hospital, Taichung, Taiwan; b Department of Internal Medicine, Faculty of Medicine, Institute of Clinical Medicine, Cardiovascular Research Center, National Yang-Ming University School of Medicine, Taipei, Taiwan; c Department of Data Science and Big Data Analytics, Providence University, Taichung, Taiwan; d Department of Financial Engineering, Providence University, Taichung, Taiwan; e Department of Medical Research, Taichung Veterans General Hospital, Taichung, Taiwan; f Department of Internal Medicine, Taichung Veterans General Hospital Chiayi Branch, Chiayi, Taiwan; g Division of Cardiology, Department of Medicine, Taipei Veterans General Hospital, Taipei, Taiwan

## Abstract

The data relates to the cohort of patients with atrial fibrillation (AF) from the National Health Insurance Research Database of Taiwan, “Rhythm Control Better Prevents Stroke and Mortality than Rate Control Strategies in Patients with Atrial Fibrillation - A Nationwide Cohort Study” (Weng et al., in press). The AF patients might receive either rate or rhythm control strategy according to the medication used. The baseline medication in rate and rhythm control groups was included in this dataset. Multivariate Cox hazards regression model was used to evaluate the hazard ratio (HR) for major adverse cardiovascular events (MACE), including ischemic/hemorrhagic stroke and mortality in AF patients receiving rate or rhythm control. The occurrence of MACE was identified from the ICD-9 CM codes. The data also contains the HR for MACE stratified by the CHA2DS2-VASc score, baseline characteristics, and the duration of strategy employed of the AF patients.

**Specifications table**

**Table:** 

Subject area	*Cardiology*
More specific subject area	*Atrial fibrillation on stroke and mortality*
Type of data	*Tables and figures*
How data was acquired	*Data analysis from the National Health Insurance Research Database of Taiwan*
Data format	*Analyzed*
Experimental factors	*Atrial fibrillation patients receiving either rate or rhythm control strategy on cardiovascular outcome*
Experimental features	*Retrospective, observational, nationwide, and population-based cohort of patients with atrial fibrillation*
Data source location	*National Health Insurance Research Database of Taiwan*
Data accessibility	*The analyzed data is with this article.*
Related research article	Weng CJ, Li CH, Liao YC, *et al.* Rhythm Control Better Prevents Stroke and Mortality than Rate Control Strategies in Patients with Atrial Fibrillation - A Nationwide Cohort Study. *Int J Cardiol* 2018 (in press) [1]

**Value of the data**

• This data provides the researchers to compare the different therapeutic strategies on cardiovascular outcomes in AF patients.
• The data has a real-world long-term cardiovascular outcome in AF patients undergoing different control strategies.
• Subgroup analysis data identifies risk factors contributing to favorable/detrimental outcomes in AF patients and helps to find out the patients at risk.

## 1 Data

**Fig. 1:**
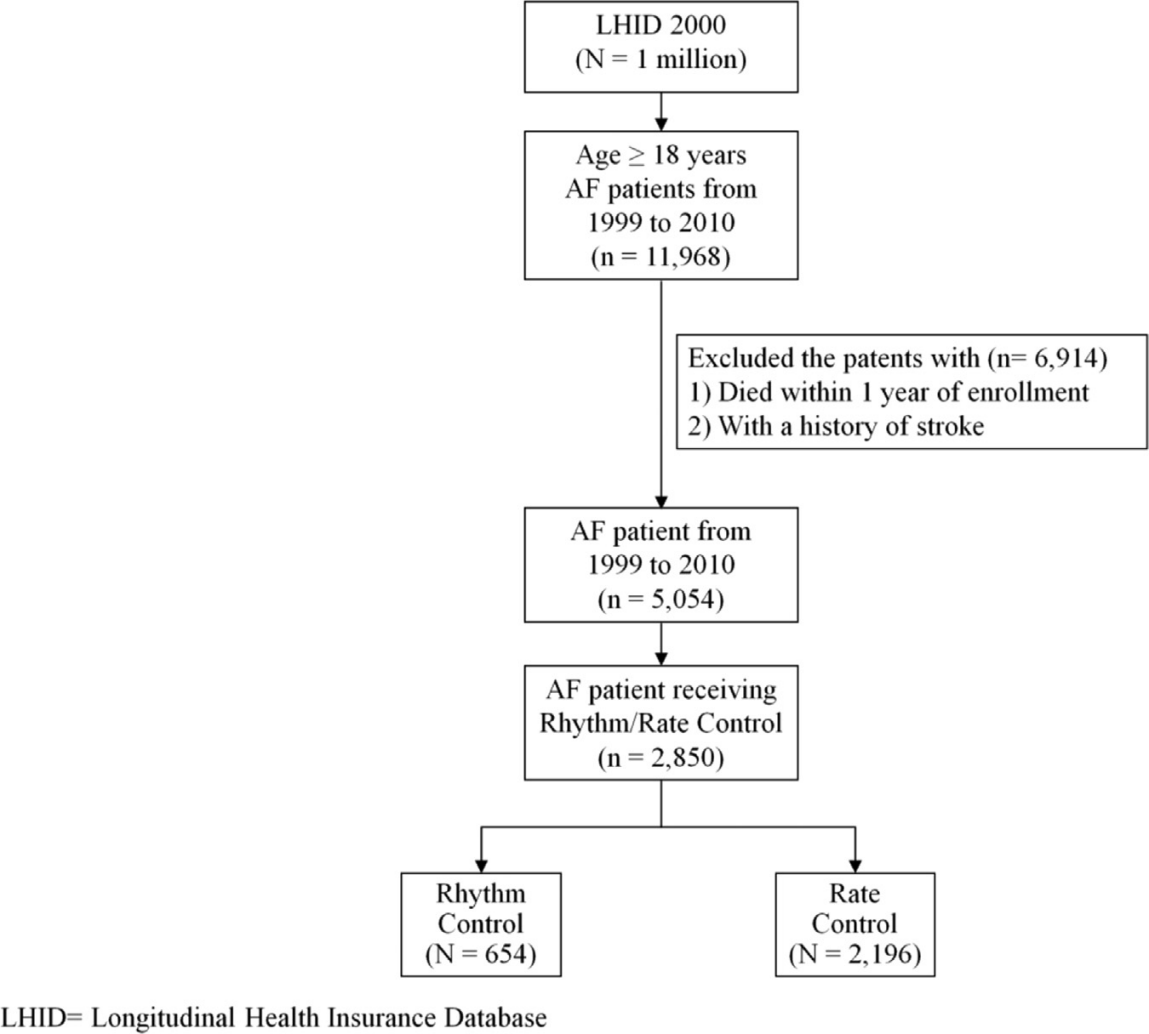
Flow chart of the AF cohort. AF, atrial fibrillation; LHID, longitudinal health insurance database.

**Fig. 2:**
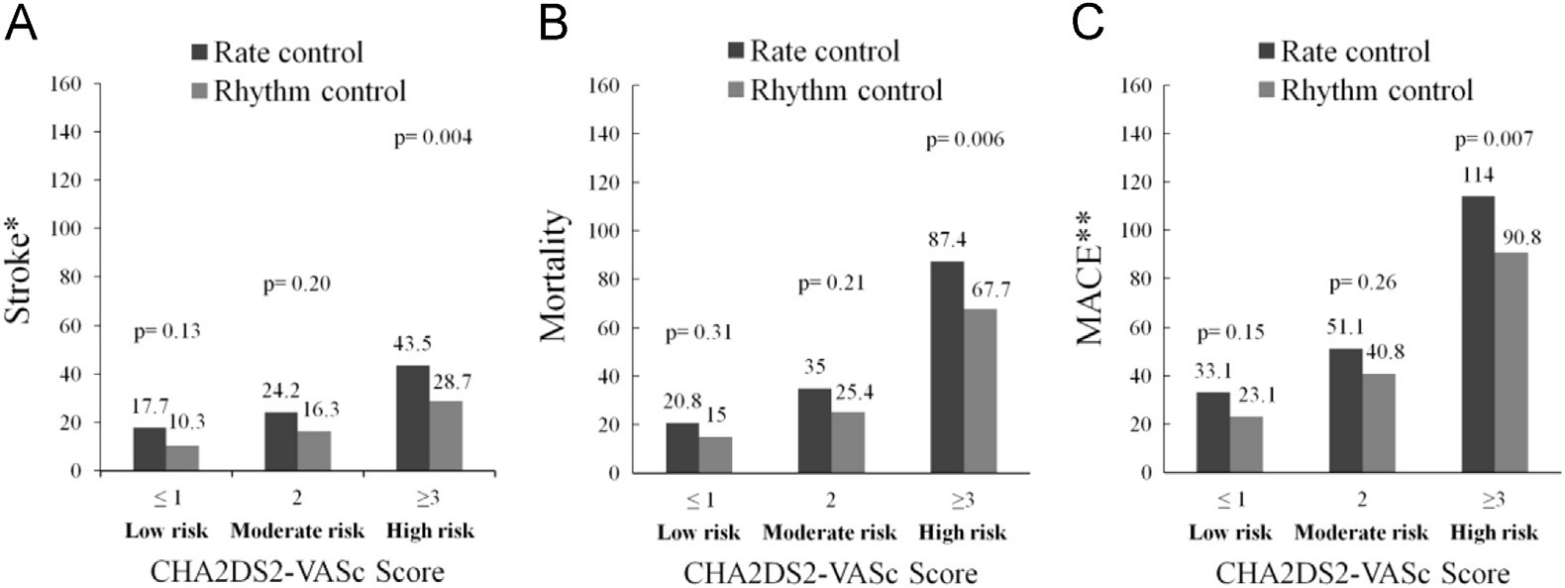
The risk of stroke (A), mortality (B), and MACE (C) between rate and rhythm control groups in low (CHA2DS2-VASc score≤1), intermediate (CHA2DS2-VASc score=2), and high (CHA2DS2-VASc score≥3) risk patients. MACE, major adverse cardiovascular event. * included ischemic and hemorrhagic stroke; ** included ischemic/hemorrhagic stroke and mortality.

Taiwan National Health Insurance program started in 1995. In this program, over 99% of the Taiwanese population (~23 million) is enrolled. The National Health Insurance Research Database of Taiwan includes records of outpatient visits, hospital admissions, prescriptions, and disease diagnoses, and is managed by the Taiwan National Health Research Institute (NHRI) [2], [3]. This data set contains AF patients retrieving from the National Health Insurance Research Database. AF patients receiving either rate or rhythm control strategies constitute the AF cohort (data set Fig. 1). The data of AF patients receiving rate versus rhythm control on major adverse cardiovascular event (MACE) stratified by CHA2DS2-VASc score is shown in Fig. 2. The medication data used in this AF cohort is shown in Table 1. Subgroup analysis data of the hazard ratio for stroke and death in this AF cohort are shown in the data set Table 2A, Table 2B, respectively. The data of the hazard ratio for stroke, death and MACE by the rate/rhythm control duration is shown in Table 3.

**Table 1:** Medications used for rate and rhythm control in patients with AF.

**Medications**	**Rate control**	**Rhythm control**	***p*-Value**
	***N* = 2196 (%)**	***N* = 654 (%)**	
Rate control			
β-blocker	1404 (63.9)	295 (45.1)	<0.0001
Diltiazem	824 (37.5)	127 (19.4)	<0.0001
Verapamil	309 (14.1)	59 (9.02)	0.0007
Digoxin	1507 (68.6)	142 (21.7)	<0.0001
Rhythm control			
Quinidine		5 (0.76)	
Flecainide		6 (0.92)	
Propafenone		277 (42.4)	
Amiodarone		481 (73.6)	
Sotalol		9 (1.38)	
Cardiovascular medication			
ACEI/ARB	1391 (63.3)	299 (45.7)	<0.0001
α-blocker	311 (14.2)	87 (13.3)	0.58
Diuretics	1399 (63.7)	255 (39.0)	<0.0001
Fibrates	111 (5.05)	29 (4.43)	0.52
Statin	296 (13.5)	96 (14.7)	0.43
Anti-thrombotics			
Aspirin	1393 (63.4)	429 (65.6)	0.31
Clopidogrel	188 (8.6)	74 (11.3)	0.03
Warfarin	420 (19.1)	81 (12.4)	<0.0001

ACEI, angiotensin converting enzyme inhibitor; ARB, angiotensin receptor blocker.

**Table 2A:** Subgroup analysis of the hazard ratio for stroke in AF patients.

Variable	Rate control		Rhythm control		Adjusted HR (95% CI)	*p*-Value	*p* for interaction
	Event	Rate	Event	Rate			
Age^a^							0.62
<65	138	24.0	27	13.3	0.65 (0.42–0.99)	0.04	
≥65	305	45.7	51	29.0	0.68 (0.50–0.92)	0.01	
Sex^b^							0.10
Female	199	36.5	35	27.3	0.80 (0.56–1.16)	0.24	
Male	244	35.0	43	17.2	0.60 (0.43–0.83)	0.002	
CHA2DS2-VASc^c^							0.99
≤1	32	17.7	12	10.3	0.61 (0.31–1.20)	0.15	
2	64	24.2	12	16.3	0.66 (0.35–1.23)	0.19	
≥3	347	43.5	54	28.7	0.66 (0.49–0.88)	0.005	
Aspirin^d^							0.70
No	149	30.7	25	18.9	0.72 (0.46–1.10)	0.13	
Yes	294	38.9	53	21.6	0.67 (0.49–0.90)	0.007	
Clopidogrel^d^							0.15
No	416	35.8	68	19.6	0.65 (0.50–0.84)	0.001	
Yes	27	33.1	10	32.6	0.91 (0.43–1.94)	0.81	
Warfarin^d^							0.89
No	354	35.4	67	20.4	0.69 (0.53–0.91)	0.007	
Yes	89	36.7	11	22.0	0.65 (0.35–1.23)	0.19	

a Model adjusted for sex, heart failure, hypertension, DM, hyperlipidemia, COPD, CKD, liver disease and peripheral vascular disease

b Model adjusted for age, heart failure, hypertension, DM, hyperlipidemia, COPD, CKD, liver disease and peripheral vascular disease

c Model adjusted for hyperlipidemia, COPD, CKD and liver disease

d Model adjusted for age, sex, heart failure, hypertension, DM, hyperlipidemia, COPD, CKD, liver disease and peripheral vascular disease

**Table 2B:** Subgroup analysis of the hazard ratio for death in AF patients.

Variable	Rate control		Rhythm control		Adjusted HR (95% CI)	*p*-Value	*p* for interaction
	Event	Rate	Event	Rate			
Age^a^							0.19
<65	177	28.1	33	15.6	0.67 (0.46–0.98)	0.04	
≥65	747	100	145	74.7	0.82 (0.68–0.98)	0.03	
Sex^b^							0.005
Female	385	63.8	77	55.3	0.95 (0.74–1.23)	0.72	
Male	539	69.6	101	38.0	0.70 (0.57–0.87)	0.002	
CHA2DS2-VASc^c^							0.99
≤1	40	20.8	18	15.0	0.83 (0.47–1.46)	0.52	
2	101	35.0	20	25.4	0.75 (0.47–1.22)	0.25	
≥3	783	87.4	140	67.7	0.78 (0.65–0.93)	0.006	
Aspirin^d^							0.02
No	327	61.7	72	51.3	0.96 (0.74–1.24)	0.75	
Yes	597	70.4	106	40.0	0.74 (0.60–0.91)	0.005	
Clopidogrel^d^							0.26
No	837	64.8	150	40.3	0.77 (0.64–0.92)	0.004	
Yes	87	102	28	83.6	1.00 (0.64–1.55)	0.99	
Warfarin^d^							0.46
No	758	68.5	160	45.5	0.85 (0.71–1.01)	0.06	
Yes	166	61.4	18	33.7	0.60 (0.37–0.98)	0.04	

a Model adjusted for sex, heart failure, hypertension, DM, hyperlipidemia, COPD, CKD, liver disease and peripheral vascular disease.

b Model adjusted for age, heart failure, hypertension, DM, hyperlipidemia, COPD, CKD, liver disease and peripheral vascular disease.

c Model adjusted for hyperlipidemia, COPD, CKD and liver disease.

d Model adjusted for age, sex, heart failure, hypertension, DM, hyperlipidemia, COPD, CKD, liver disease and peripheral vascular disease.

**Table 3:** Hazard ratio for stroke, death and MACE by the rate/rhythm control duration.

AF control Strategy	Adjusted HR (95% CI)		
	Stroke	Mortality	MACE
Rate control	Ref	Ref	Ref
Rhythm control			
30–179 cDDD	0.74 (0.53–1.02)	0.91 (0.74–1.13)	0.93 (0.77–1.13)
180–364 cDDD	0.77 (0.54–1.12)	0.73 (0.56–0.95)*	0.79 (0.63–1.00)*
≥365 cDDD	0.34 (0.16–0.72)*	0.68 (0.45–1.02)	0.60 (0.41–0.87)*

Model adjusted for age, sex, heart failure, hypertension, DM, hyperlipidemia, COPD, CKD, liver disease and peripheral vascular disease

The duration of rate/rhythm control was stratified by cumulative defined daily doses (cDDDs) of the rate/rhythm control medication used.

* <0.05

## 2 Experimental design, materials and methods

### 2.1 Research database

The data set was created by a systemic randomized sampling of 1,000,000 patients from 1999 to 2010 in the National Health Insurance Research Database. This data set has been was confirmed to be representative of the general Taiwanese population. Since the patient׳s data was provided in an anonymous format, the written informed consents were waived. The creation of this data set was approved by the Institutional Review Board of Taichung Veterans General Hospital (CE13152B-4).

### 2.2 Patient population

To create the AF cohort data set, patients aged ≥ 18 years with a diagnosis of atrial flutter/fibrillation (AF), were identified by the International Classification of Diseases, 9th Revision, Clinical Modification (ICD-9-CM) codes 427.3, 427.31, and 427.32. The diagnosis of AF was defined as three or more outpatient visits with a diagnostic code of AF within a year, or at least one hospitalization under an AF diagnostic code. The primary endpoints of the AF cohort were MACE, including hemorrhagic stroke (ICD-9-CM: 430-432), ischemic stroke (ICD-9-CM: 433–438), and death, therefore, patients were excluded from this cohort if they had experienced prior stroke or had died within one year of enrollment.

### 2.3 Definitions of medication use

Patients who had used any one of the anti-arrhythmic drug (AADs) for AF rhythm control, and had a defined daily dose (DDD) of ≥30 within the first year of enrollment, were defined as the rhythm control group. The AADs and their classes for AF rhythm control included amiodarone (III), sotalol (III), propafenone (Ic), flecainide (Ic), quinidine (Ia), and procainamide (Ia). AF patients who received any rate control medications, including beta-blockers, calcium channel blockers (diltiazem, verapamil), and digitalis for ≥30 DDD within the first year of enrollment constituted the rate control group. Patients who used both rhythm and rate control medications were classified as the rhythm control group. AF treatment strategies in this cohort were chosen by physicians’ clinical discretion. Current use was defined as taking medication between the prescription date and the end date of drug supply. Discontinuation was defined as when no medication was refilled after the end date of drug prescription. The data set also contains commonly prescribed antithrombotic therapies, including warfarin, acetylsalicylic acid, and clopidogrel for analysis.

### 2.4 Outcomes and covariates

The baseline demographic data was recorded. Cardiovascular co-morbidities including hypertension, hyperlipidemia, liver disease, diabetes mellitus (DM), coronary heart disease (CHD), congestive heart failure (CHF), peripheral vascular disease (PVD), valvular heart disease (VHD), chronic obstructive pulmonary disease (COPD), and chronic kidney disease (CKD) were identified by the ICD-9-CM diagnostic code if the patient had at least 1 hospitalization or 3 consecutive outpatient visits under the diagnosis of the above listed diseases.

### 2.5 Statistical analysis

Continuous variables were presented as mean ± standard deviations (SD), while proportions were used for categorical variables. Analysis of variance and Chi-square tests were used for comparing differences in the continuous and categorical variables. Multivariable Cox proportional hazard regression models were used to exclude confounding factors contributing to MACE occurrence (adjusted for age, gender, co-morbidities, and medications). A stratified analysis was used to evaluate the cardiovascular outcomes in patients with/without the specified medications. The rate control group served as the reference, and the occurrence of MACE in the rhythm control group was expressed by the hazard ratio (HR) and a 95% confidence interval (CI). All statistical analyses were carried out using SAS software version 9.2 (SAS Institute, Inc., Cary, NC, USA). A p value of <0.05 was considered statistically significant.
