# Supplementary material for: Data for rate versus rhythm control strategy on stroke and mortality in patients with atrial fibrillation
Source: Data Brief. 2018 Sep 6;20:1279–85. doi: 10.1016/j.dib.2018.08.199 (PMC6143751; doi:10.1016/j.dib.2018.08.199)
Supplement: Supplementary file 1 — Supplementary material [file mmc1.pdf]

AUTHOR DECLARATION FORM

Manuscript title：**Data for Rate versus Rhythm Control Strategy on Stroke and Mortality in Patients with Atrial Fibrillation**

We confirm that there are no known conflicts of interest associated with this publication and there has been no significant financial support for this work that could have influenced its outcome.

We confirm that the manuscript has been read and approved by all named authors and that there are no other persons who satisfied the criteria for authorship but are not listed. We further confirm that the order of authors listed in the manuscript has been approved by all of us.

We confirm that we have given due consideration to the protection of intellectual property associated with this work and that there are no impediments to publication, including the timing of publication, with respect to intellectual property. In so doing we confirm that we have followed the regulations of our institutions concerning intellectual property.
We further confirm that any aspect of the work covered in this manuscript that has involved either experimental animals or human patients has been conducted with the ethical approval of all relevant bodies and that such approvals are acknowledged within the manuscript.

We understand that the Corresponding Author is the sole contact for the Editorial process (including Editorial Manager and direct communications with the office). He/she is responsible for communicating with the other authors about progress, submissions of revisions and final approval of proofs. We confirm that we have provided a current, correct email address which is accessible by the Corresponding Author and which has been configured to accept email from **ychsieh@vghtc.gov.tw**

Signed by all authors as follows:

Print Name	Signature	Date
Chi-Jen Weng	Chi-Jen, Weng	06/27/2018
Cheng-Hung Li	Cheng-Hung. Li	06/26/2018
Ying-Chieh Liao	Ying-Chieh Liao	06/26/2018
Che-Chen Lin	Che-Chun Lin	06/27/2018
Jiunn-Cherng Lin		
Shih-Lin Chang		
Chu-Pin Lo		
Kuo-Ching Huang		
Jin-Long Huang	[illegible]	06/27/2018
Ching-Heng Lin	Ching-Heng Lin	06/27/2018
Yu-Cheng Hsieh	Yu-Cheng Hsieh	06/26/2018
Tsu-Juey Wu	Tsu-Juey Wu	06/26/2018

AUTHOR DECLARATION FORM

Manuscript title：**Data for Rate versus Rhythm Control Strategy on Stroke and Mortality in Patients with Atrial Fibrillation**

We confirm that there are no known conflicts of interest associated with this publication and there has been no significant financial support for this work that could have influenced its outcome.

We confirm that the manuscript has been read and approved by all named authors and that there are no other persons who satisfied the criteria for authorship but are not listed. We further confirm that the order of authors listed in the manuscript has been approved by all of us.

We confirm that we have given due consideration to the protection of intellectual property associated with this work and that there are no impediments to publication, including the timing of publication, with respect to intellectual property. In so doing we confirm that we have followed the regulations of our institutions concerning intellectual property.
We further confirm that any aspect of the work covered in this manuscript that has involved either experimental animals or human patients has been conducted with the ethical approval of all relevant bodies and that such approvals are acknowledged within the manuscript.

We understand that the Corresponding Author is the sole contact for the Editorial process (including Editorial Manager and direct communications with the office). He/she is responsible for communicating with the other authors about progress, submissions of revisions and final approval of proofs. We confirm that we have provided a current, correct email address which is accessible by the Corresponding Author and which has been configured to accept email from **ychsieh@vghtc.gov.tw**

Signed by all authors as follows:

Print Name	Signature	Date
**Chi-Jen Weng**		
**Cheng-Hung Li**		
**Ying-Chieh Liao**		
**Che-Chen Lin**		
**Jiunn-Cherng Lin**	Lin Jiunncherng	2017. Jun. 7th
**Shih-Lin Chang**		
**Chu-Pin Lo**		
**Kuo-Ching Huang**		
**Jin-Long Huang**		
**Ching-Heng Lin**		
**Yu-Cheng Hsieh**		
**Tsu-Juey Wu**		

AUTHOR DECLARATION FORM

Manuscript title：**Data for Rate versus Rhythm Control Strategy on Stroke and Mortality in Patients with Atrial Fibrillation**

We confirm that there are no known conflicts of interest associated with this publication and there has been no significant financial support for this work that could have influenced its outcome.

We confirm that the manuscript has been read and approved by all named authors and that there are no other persons who satisfied the criteria for authorship but are not listed. We further confirm that the order of authors listed in the manuscript has been approved by all of us.

We confirm that we have given due consideration to the protection of intellectual property associated with this work and that there are no impediments to publication, including the timing of publication, with respect to intellectual property. In so doing we confirm that we have followed the regulations of our institutions concerning intellectual property.
We further confirm that any aspect of the work covered in this manuscript that has involved either experimental animals or human patients has been conducted with the ethical approval of all relevant bodies and that such approvals are acknowledged within the manuscript.

We understand that the Corresponding Author is the sole contact for the Editorial process (including Editorial Manager and direct communications with the office). He/she is responsible for communicating with the other authors about progress, submissions of revisions and final approval of proofs. We confirm that we have provided a current, correct email address which is accessible by the Corresponding Author and which has been configured to accept email from **ychsieh@vghtc.gov.tw**

Signed by all authors as follows:

Print Name	Signature	Date
**Chi-Jen Weng**		
**Cheng-Hung Li**		
**Ying-Chieh Liao**		
**Che-Chen Lin**		
**Jiunn-Cherng Lin**		
**Shih-Lin Chang**	Shih-Lin Chang	2018/6/26
**Chu-Pin Lo**		
**Kuo-Ching Huang**		
**Jin-Long Huang**		
**Ching-Heng Lin**		
**Yu-Cheng Hsieh**		
**Tsu-Juey Wu**		

AUTHOR DECLARATION FORM

Manuscript title：**Data for Rate versus Rhythm Control Strategy on Stroke and Mortality in Patients with Atrial Fibrillation**

We confirm that there are no known conflicts of interest associated with this publication and there has been no significant financial support for this work that could have influenced its outcome.

We confirm that the manuscript has been read and approved by all named authors and that there are no other persons who satisfied the criteria for authorship but are not listed. We further confirm that the order of authors listed in the manuscript has been approved by all of us.

We confirm that we have given due consideration to the protection of intellectual property associated with this work and that there are no impediments to publication, including the timing of publication, with respect to intellectual property. In so doing we confirm that we have followed the regulations of our institutions concerning intellectual property.
We further confirm that any aspect of the work covered in this manuscript that has involved either experimental animals or human patients has been conducted with the ethical approval of all relevant bodies and that such approvals are acknowledged within the manuscript.

We understand that the Corresponding Author is the sole contact for the Editorial process (including Editorial Manager and direct communications with the office). He/she is responsible for communicating with the other authors about progress, submissions of revisions and final approval of proofs. We confirm that we have provided a current, correct email address which is accessible by the Corresponding Author and which has been configured to accept email from **ychsieh@vghtc.gov.tw**

Signed by all authors as follows:

Print Name	Signature	Date
Chi-Jen Weng		
Cheng-Hung Li		
Ying-Chieh Liao		
Che-Chen Lin		
Jiunn-Cherng Lin		
Shih-Lin Chang		
Chu-Pin Lo	Chu-Pin Lo	2018. 6. 27
Kuo-Ching Huang		
Jin-Long Huang		
Ching-Heng Lin		
Yu-Cheng Hsieh		
Tsu-Juey Wu		

AUTHOR DECLARATION FORM

Manuscript title：**Data for Rate versus Rhythm Control Strategy on Stroke and Mortality in Patients with Atrial Fibrillation**

We confirm that there are no known conflicts of interest associated with this publication and there has been no significant financial support for this work that could have influenced its outcome.

We confirm that the manuscript has been read and approved by all named authors and that there are no other persons who satisfied the criteria for authorship but are not listed. We further confirm that the order of authors listed in the manuscript has been approved by all of us.

We confirm that we have given due consideration to the protection of intellectual property associated with this work and that there are no impediments to publication, including the timing of publication, with respect to intellectual property. In so doing we confirm that we have followed the regulations of our institutions concerning intellectual property.
We further confirm that any aspect of the work covered in this manuscript that has involved either experimental animals or human patients has been conducted with the ethical approval of all relevant bodies and that such approvals are acknowledged within the manuscript.

We understand that the Corresponding Author is the sole contact for the Editorial process (including Editorial Manager and direct communications with the office). He/she is responsible for communicating with the other authors about progress, submissions of revisions and final approval of proofs. We confirm that we have provided a current, correct email address which is accessible by the Corresponding Author and which has been configured to accept email from **ychsieh@vghtc.gov.tw**

Signed by all authors as follows:

Print Name	Signature	Date
**Chi-Jen Weng**		
**Cheng-Hung Li**		
**Ying-Chieh Liao**		
**Che-Chen Lin**		
**Jiunn-Cherng Lin**		
**Shih-Lin Chang**		
**Chu-Pin Lo**		
**Kuo-Ching Huang**	K.C. [illegible]	2018.6.27.
**Jin-Long Huang**		
**Ching-Heng Lin**		
**Yu-Cheng Hsieh**		
**Tsu-Juey Wu**		
